# Rethinking Goods and Services Tax (GST) and Academic Research in India

**DOI:** 10.7759/cureus.109259

**Published:** 2026-05-20

**Authors:** Venkataramana Kandi

**Affiliations:** 1 Clinical Microbiology, Prathima Institute of Medical Sciences, Karimnagar, IND

**Keywords:** goods and services tax (gst), health policy, india, innovation, medical education, public health, research, taxation, universities

## Abstract

The Goods and Services Tax (GST), introduced in India on July 1, 2017, was envisioned as a landmark reform to unify indirect taxation, enhance transparency, and reinforce fiscal federalism. While celebrated for its efficiency, its uniform application to research‑related activities has generated significant concern across academia, medicine, and public health. Conference registration fees and continuing medical education (CME) costs have risen sharply, restricting opportunities for skill development among doctoral students, postdoctoral fellows, clinicians, and public health professionals. Escalating publication charges and subscription cancellations in state universities, teaching hospitals, and research institutes have curtailed access to international medical literature, limiting evidence‑based practice, policy formulation, and equitable knowledge dissemination. Procurement delays in national laboratories and medical research centers further impede innovation, slowing translation of discoveries into improved diagnostics, therapeutics, and preventive strategies. From the government’s perspective, uniform taxation prevents loopholes, curbs misuse of exemptions, and ensures consistency in revenue collection, with GST revenues earmarked for infrastructure, healthcare, and education. Yet, the imposition of an 18% GST on research and CME activities clashes with the lived experiences of researchers and healthcare professionals, who view it as undermining accessibility, equity, and timely adoption of global best practices. The downstream effects include weakened epidemic preparedness, delayed integration of innovations into healthcare delivery, and widening disparities in public health outcomes. Formal petitions from scientific academies, medical associations, industry bodies, and state assemblies underscore the urgency of reform. International comparisons reveal alternative fiscal models that balance efficiency with recognition of research and CME as public goods. This editorial argues for targeted reforms, such as concessional rates, exemptions for academic medical services, and sector‑specific subsidies, to align fiscal policy with India’s innovation and public health goals, ensuring taxation does not become a barrier to advancing healthcare equity and outcomes.

## Editorial

The Goods and Services Tax (GST), introduced in India on July 1, 2017, was intended to unify the indirect tax system, eliminate cascading levies, and strengthen fiscal federalism. By replacing excise duties, service taxes, and state‑level charges, GST aimed to enhance transparency, broaden the tax base, and generate stable revenues. From the government’s perspective, GST is a modern mechanism for revenue collection, ensuring uniformity and fiscal sustainability [1].

However, applying GST at 18% to research‑related activities, including conferences, workshops, journal publications, and procurement of scientific equipment, has provoked debate. Researchers argue these are essential platforms for knowledge dissemination, not commercial enterprises. Institutions, particularly state universities and medical colleges, report financial strain, procurement delays, and reduced access to journals, while smaller colleges struggle with compliance. The academic community views GST as undermining accessibility and equity, widening the gap between elite institutions and regional universities [2].

For researchers, GST has raised barriers. Conference fees have risen nearly 40%, discouraging participation among doctoral and postdoctoral fellows [3]. Open‑access publication charges increased from ₹35,000 to ₹55,000 per paper [3,4]. Libraries, especially at state universities, canceled subscriptions to international journals. Policy analyses note that consortium access under the E‑ShodhSindhu scheme faced funding pressures between 2017 and 2022, with estimates suggesting 35-40% of subscriptions were discontinued. While GST contributed to rising costs, attributing cancellations solely to taxation requires caution, as broader budgetary constraints also played a role [5].

Medical researchers face added challenges: continuing medical education (CME) programs, clinical workshops, and international databases are taxed at commercial rates, restricting evidence‑based practice in infectious diseases, maternal health, and antimicrobial resistance. Under GST 2.0 reforms, essential medical devices, such as dialysis machines, pacemakers, and orthopedic supports, were shifted from the 12-18% slab to a concessional 5% rate, reducing treatment costs [6]. Health insurance premiums for individuals, including family floater and senior citizen policies, were moved to the nil‑rated category (0%), though group insurance policies remain taxed at 18% [7]. These changes have eased some burdens on patients, but academic services, such as conferences, workshops, and journal publications, continue to attract 18% GST, leaving research institutions exposed to significant financial strain.

Empirical data highlight broader consequences. India loses approximately $10 billion annually in foreign exchange due to students studying abroad, while skilled professional migration costs the economy an estimated $2 billion per year [8,9]. Rising academic costs, including GST‑linked conference and publication fees, amplify these pressures, contributing to brain drain. Equity concerns are acute: elite institutions absorb costs, while smaller colleges face disproportionate burdens, widening systemic disparities. Public health suffers as CME programs taxed at commercial rates restrict evidence‑based practice against infectious diseases and antimicrobial resistance [6,7].

Institutions face financial and administrative strain. Public universities divert funds from infrastructure and faculty recruitment to meet tax obligations, while rural colleges lack resilience. Procurement timelines for laboratory instruments have extended from two to seven months; the Council for Scientific and Industrial Research (CSIR) confirmed in May 2023 that withdrawal of GST concessions complicated contracts and delayed deliveries [2,3,10]. Libraries have canceled subscriptions, weakening publication opportunities. Administrative complexity compounds the problem: universities must file GST returns, maintain records, and undergo audits, diverting resources from academics. Smaller colleges without finance departments rely on overstretched faculty, leading to inefficiencies and penalties. Despite reforms in healthcare and insurance, the uniform 18% GST on academic services continues to equate universities with profit‑driven organizations, disregarding their public mission [7].

The government defends the uniform 18% rate on academic services, citing past misuse of exemptions by private coaching centers. In 2023-24, education contributed ₹4,792 crore in GST revenue, just 0.26% of total collections. Relief measures under GST 2.0, including nil‑rating of educational materials and health insurance premiums, have not extended to higher education services. Officials argue uniformity prevents loopholes and ensures revenue neutrality. Yet researchers and institutions see GST as a barrier to knowledge dissemination. Bodies such as the Indian National Science Academy (INSA) and the Federation of Indian Chambers of Commerce and Industry (FICCI) have petitioned the GST Council, and state assemblies in Kerala and Tamil Nadu have passed resolutions urging relief [11-13]. The government has resisted broad exemptions, maintaining the 18% rate on academic services.

International models show alternatives. Malaysia introduced GST in 2015 at 6%, applying it to academic services. Rising costs and declining participation led to repeal in 2018 and replacement with the Sales and Service Tax (SST), excluding academia. In the European Union (EU), value-added tax (VAT) rates range from 15-25%, but universities benefit from exemptions or reclaim mechanisms, minimizing the net burden. Subsidies further shield academia, allowing conferences and publications to thrive [4]. These examples demonstrate that fiscal efficiency can coexist with recognition of research as a public good.

India’s GST regime has far‑reaching consequences. Reduced participation in conferences slows innovation, journal cancellations limit global exposure, and inequities deepen systemic disparities. Risks of brain drain and widening inequities are supported by migration and healthcare cost data, underscoring taxation as a significant aggravating factor. GST’s rigid application undermines India’s competitiveness at a critical time [14].

Targeted reforms are urgently needed. Restoring concessional rates of 5% for conferences, workshops, and journals would lower barriers, especially for state universities and medical colleges. Exempting educational institutions from GST would prevent academia from being treated as commercial enterprises and align India with EU models. Research and development (R&D) tax credits could incentivize infrastructure investment, while subsidies for journal subscriptions and conference participation would promote equity. Differentiated tax slabs distinguishing academic services from commercial training would recognize research as a public good. Collectively, these measures would realign fiscal policy with national innovation goals, ensuring taxation supports rather than hinders India’s scientific ambitions (Table 1).

**Table 1:** GST impact on academic research and policy comparisons GST: goods and services tax; EU: European Union; VAT: value-added tax; SST: sales and services tax; R&D: research and development; IITs: Indian Institute of Technology; IISc: Indian Institute of Science; CME: continuing medical education

Dimension / Variable	India (pre‑GST 5%)	India (post‑GST 18%)	Observed impact in India	International comparison	Case studies	Suggested policy reforms
Conference registration fees	Approximately ₹12,000	Approximately ₹16,500	Nearly 40% increase; reduced participation among early‑career researchers and faculty from state universities [1]	EU’s VAT exemptions/reclaim minimize burden; Malaysia repealed GST in 2018 excluding academic services [4]	Indian institutions saw a 30% decline in conference attendance; exclusion of early‑career researchers [14]	Restore concessional rates to 5%
Procurement timelines	Approximately 2 months	Approximately 7 months	Delays in acquiring instruments and reagents; slowed productivity [5]	EU’s VAT reclaim avoids delays; Malaysia’s SST excluded academic imports [4]	Workshops: costs rose ~20%; reduced student participation [14]	Introduce R&D tax credits
Journal subscriptions	Full access maintained	28% decline	Cancellations concentrated in medical/engineering databases; weakened access to global knowledge [6]	EU subsidies and VAT relief sustain access; Malaysia's repeal restored affordability [4]	University libraries saw 28% decline in subscriptions; cancellations in medical/engineering journals [4]	Provide subsidies for journal access
Equity in institutions	Balanced between elite and regional	Elite absorb costs; regional/state universities strained [14]	Widened gap between IITs/IISc and smaller universities; risk of brain drain [4]	EU’s equitable access via exemptions; Malaysia's repeal reduced inequities [4]	Inequity between elite and regional universities noted in workshops, conferences, and journal publications [4]	Exempt educational institutions from GST
Medical education and public health	CME programs affordable	CME programs/workshops taxed at commercial rates [6]	Restricted evidence‑based practice; downstream effects on infectious diseases, maternal health, antimicrobial resistance [6]	EU’s VAT exemptions protect medical education; Malaysia's SST excluded academic health services [4]	Medical college CME’s unaffordable; reduced junior doctor participation [5]	Differentiated tax slabs for academic vs. commercial services
Policy alignment	Research recognized as public good	Research treated as taxable commerce [1]	Contradicts India’s ambition to be global leader in science/technology [14]	EU balances fiscal efficiency with academic relief; Malaysia’s responsive repeal after opposition [4]	Malaysia’s (2015–2018) 6% GST raised costs; repealed in 2018; EU has high VAT, but universities reclaim [4]	Comprehensive reform package combining concessional rates, exemptions, credits, subsidies

In conclusion, India's GST highlights contradictions between fiscal efficiency and the public good of research. Although GST 2.0 revisions brought assistance by exempting health insurance premiums and instructional materials and lowering medical device taxation to 5%, academic services, including as conferences, workshops, and journal publications, are still taxed at 18%. This has increased participation obstacles, widened disparities between elite and regional institutions, and hindered innovation, with smaller universities and medical colleges bearing a disproportionate burden. Rising prices contribute to student migration, professional brain drain, and limited access to CME, compromising public health readiness. International examples, such as Malaysia's abolition of GST for academia and the European Union's VAT exemptions, show potential alternatives. Therefore, it is strategically necessary to reform GST in India. Restoring concessional rates, exempting educational institutions, and implementing R&D tax credits will better connect fiscal policy with the country's innovation objectives. Without these steps, India runs the risk of losing its capacity for public health, its research ecosystem, and its ability to compete globally.
